# The Utilization of a Pregnancy-Associated Glycoprotein Profile and PAG/P4 Ratio Biomarker for the Diagnosis of Pseudopregnancy in Dairy Goats

**DOI:** 10.3390/vetsci11110574

**Published:** 2024-11-16

**Authors:** Carlos Cruz, Gisele Margatho, Miguel Simões, João Simões

**Affiliations:** 1 Department of Veterinary Sciences, Vasco da Gama University School, Avenida José R. Sousa Fernandes, 197 Lordemão, 3020-210 Coimbra, Portugal; carlos.cruz@euvg.pt; 2 Department of Veterinary Sciences, Veterinary and Animal Research Centre (CECAV), AL4AnimalS, School of Agricultural and Veterinary Sciences, University of Trás-os-Montes and Alto Douro (UTAD), 5000-801 Vila Real, Portugal; giselem@utad.pt; 3 Department of Veterinary Sciences, Cruzvet—Medicina e Produção, Lda., 3045-480 Coimbra, Portugal; mhsimoes@outlook.pt

**Keywords:** hydrometra, goats, PAGs, progesterone, biomarker, reproductive disorders

## Abstract

Pseudopregnancy (PPG) is a common reproductive disorder in dairy goats, characterized by the accumulation of sterile fluid in the uterus and persistence of a corpus luteum, leading to temporary infertility and reduced farm efficiency. This study aimed to determine the pregnancy-associated glycoprotein (PAG) profile in dairy goats and to evaluate the PAG/P4 ratio as a potential novel biomarker for PPG diagnosis. A total of 605 Saanen and crossbreed mated goats were evaluated via transabdominal ultrasonography between 31 and 52 days after the removal of bucks in four intensive dairy farms, presenting an overall PPG incidence of 7.8%. Blood samples from PPG (n = 47), nonpregnant (NPG; negative control; n = 11), and pregnant (PG; positive control; n = 10) does were collected for PAG and P4 analysis. The PAG plasma levels were 0.08 ± 0.02, 0.13 ± 0.04, and 1.45 ± 0.04 OD (optical density) in the PPG, NPG, and PG groups, respectively (*p* < 0.001). The PAG/P4 ratio was lower in the PPG group (0.01 ± 0.11; *p* < 0.001) than in the NPG (0.24 ± 0.23) and PG (0.18 ± 0.23) groups. This difference allows for the establishment of a new methodological diagnostic approach to PPG. The area under the curve (AUC) was 0.996 for a cut-off of 0.04 and 97.9% and 100% sensitivity and specificity, respectively. This study suggests that the PAG/P4 ratio can serve as a reliable biomarker for PPG diagnosis, helping to distinguish it from pregnancy and nonpregnancy and improving reproductive management in dairy goats.

## Simple Summary

A total of 47 pseudopregnant (PPG), 11 nonpregnant (NPG), and 10 pregnant (PG) does were used to characterize the profile of pregnancy-associated glycoproteins (PAGs) and evaluate the PAG/P4 ratio as a feasible biomarker to detect pseudopregnancy (PPG) in four intensive dairy farms. At 34.5 ± 0.5 days after the removal of bucks, uterine ultrasonographic examination and blood sample collection were performed in all groups. A PAG/P4 ratio cut-off of 0.04 was obtained for a sensitivity and specificity of 97.9 and 100%, respectively, and an area under the curve (AUC) of 0.996. This novel ratio biomarker could become a feasible tool to diagnose pseudopregnancy in intensive dairy goat farms and should be tested in a large goat population.

## 1. Introduction

Pseudopregnancy (PPG), or hydrometra, is a reproductive disorder that is frequently found in dairy goats [1]. The PPG incidence and prevalence in farms vary between studies due to different risk factors [2,3]. Nonetheless, a prevalence of approximately 10% (904/8642) was reported in a long-term study (1987–2015) on two UK dairy goat farms (Alpine, Saanen, and Toggenburg breeds) [2]. Over the past 10 years, the prevalence and incidence of PPG in dairy goat farms have remained relevant. A mean annual PPG incidence of 17% (95% CI: 14 to 21%) was reported (through a questionnaire aimed at farmers and veterinarians) in Dutch dairy goat farms [3]; a mean prevalence of 10% (268/2680) was also observed in Brazilian dairy goat farms, mainly in Alpine, Saanen, and Toggenburg breeds [4]; and a 9% (118/1311) prevalence was observed in the Murciano-Granadina breed on an Iranian farm [5].

PPG is characterized by an abnormal intrauterine accumulation of sterile fluid and the persistence of a functional corpus luteum (CL) [6], which is responsible for keeping the cervix closed, and a pathological anestrus. One of the relevant causes for non-return to estrus is PPG. This temporary infertility increases the birth interval, decreasing milk production and farms’ reproductive efficiency [1,7,8].

A failure to return to cyclical activity and gradual abdominal distension lead to the diagnosis being made during routine pregnancy B-mode ultrasonography (UTR), 30–40 days after artificial insemination (AI) or natural breeding [9,10]. To differentiate PPG from pregnancy, the examination is based on the recognition of an enlarged anechoic fluid-filled uterus in the absence of fetuses or placentomes [6,11]. PPG can be observed in goats with spontaneous or synchronized ovulation and in mated or unmated anestrus does during breeding and non-breeding seasons [8,12].

The etiology and pathophysiology of PPG are still poorly understood. Evidence indicates the presence of a persistent CL, occurring after a non-fertilized mating or due to an early or late embryonic death [8,12,13,14]. A high plasma progesterone (P4) concentration (above 1–2 ng/mL) seems to be a prerequisite for the development and maintenance of PPG. However, high P4 levels (higher than 1 ng/mL) only indicate the presence of functional CL, usually associated either with pregnancy or diestrus of cycling does but also in abnormal conditions such as a longer length of the estrous cycle, early embryonic death, luteal cysts, or PPG.

Pregnancy-associated glycoproteins (PAGs) are antigens that are secreted by the placenta and enter the maternal bloodstream around the time of implantation, being detected in the peripheral circulation of pregnant does. After being secreted by the placenta trophectoderm cells, they migrate from the fetal tissue to fuse with maternal uterine epithelial cells and form hybrid feto-maternal trinucleate cells. These cells are responsible for the release of glycoproteins in the maternal organism, presupposing the presence of functional and healthy trophoblastic tissue [15,16]. Thus, the presence of PAGs in the mother’s blood serum not only represents a useful pregnancy diagnostic tool from 21 to 24 days post conception but also provides information about embryonic and/or fetal viability [17]. In fact, PAGs allow one to predict early pregnancy failures, as their serum levels quickly decrease after embryonic death [18,19,20,21], presenting a serum half-life of about 7.5 days in goats [2]. An indirect diagnosis of embryonic mortality can be made when, after taking two blood samples, the serum PAG concentration of the second one falls below the threshold to define does as pregnant or nonpregnant. One single sample with a serum PAG concentration higher than the mentioned threshold during the return to estrus can also be conclusive.

Typically, in intensive production systems of dairy goats, breeding management is based on the formation of several groups of does, according to their days in lactation, throughout the year. This breeding management ensures a relatively constant monthly milk yield production from this seasonal species. After estrus induction (hormonal protocol, male effect, photoperiod treatment), bucks usually gather with the groups of does over a number of weeks to maximize the number of pregnant does. Normally, pregnancy and PPG are diagnosed using UTR (a cheap and practical methodology) during periodical veterinary visits to farms. González et al. 2001 [20] observed similar high accuracies of diagnosing pregnancy for both the UTR and PAG methods on days 24–26 post mating. However, they reported a lower accuracy using blood P4 levels on day 22 post mating (close to the return to estrus in nonpregnant animals) to identify nonpregnant does. In this context, the question remains whether the measurement of blood PAGs and P4 can serve as a diagnostic tool for the main reproductive events (pregnancy, nonpregnancy, and PPG) after mating under farm breeding management conditions without knowing the exact day of mating within each group of does.

Although the PAG and P4 profiles of pregnant does have been described before, the PAG profile and the ratio between PAGs and P4 have not been reported in PPG to the best of our knowledge. This potential new methodology (PAG/P4 ratio) may allow for an early diagnosis of PPG without the use of UTR or complement it. Hence, the aims of the present study were to (i) characterize the PAG profile in PPG; (ii) determine differences in plasma PAG concentration (optical density) according to the pregnancy status and PPG occurrence; and (iii) evaluate if the PAG/P4 ratio can serve as a novel biomarker for PPG diagnosis. We hypothesize that the PAG/P4 ratio biomarker can accurately differentiate PPG from PG and NPG does. This hypothesis was formulated mainly due to (a) the potential causes of PPG, i.e., subsequent embryonic death; (b) the high accuracy of PAGs as a pregnancy biomarker from approximately three weeks after conception to four weeks after kidding or pregnancy loss; and (c) the P4 profiles in PPG, NPG, and PG does.

## 2. Materials and Methods

This study was conducted in accordance with the Declaration of Helsinki. Animal management and blood collections from does were part of the breeding program and reproductive herd health plan of the farms as part of the veterinary services of Cruzvet—Medicina e Produção, Lda. (Coimbra, Portugal).

### 2.1. Animals, Management, and Sample Size

This study was performed between 13 July 2023 and 16 January 2024 in four dairy goat farms, located in the central region of Portugal (40° N 8° W and 39° N 8° W), a temperate Mediterranean climate region. The animals, Saanen and related crossbreeds, were reared under an intensive system for dairy production, fed under proper feeding schedules with a commercial balanced diet and water ad libitum. All farms were free of brucellosis, regularly dewormed, and vaccinated for enterotoxemia and contagious agalactia diseases.

Goats were confined in different pens according to their production and reproductive status and separated from bucks. In all these farms, the breeding programs were based on the establishment of goat groups for reproduction purposes every three months to ensure a stable milk yield production throughout the year. Breeding programs involved female estrous synchronization protocols, i.e., progestogen-based protocols or melatonin subcutaneous implants, followed by natural mating. Mating was achieved by the bucks’ introduction (BI) in each group, with a ratio of 1:5 or 1:15 according to anestrus or breeding season, respectively, over 31 to 52 days.

To estimate the PPG incidence in the farms, the sample size was calculated for a finite population of 700 does (expected breeding does during the study period) and according to the following equation [22,23]:

n = [Z^2^ × P × (1 − P)]/ε^2^

and adjusted for small/finite populations: n(adj) = (N × n)/(N + n).

The sample size (adjusted for finite) was n(adj) = 583 for a 95% confidence level (Z = 1.96 for α = 0.05), 1% margin of error (ε), 10% of population proportion with PPG (P) [4], and population size (N) of 700 does. A total of 605 does, i.e., all does used for breeding purposes on the four farms, were used during the study period.

### 2.2. Ultrasonographic Evaluation, Data Obtention, and Definition of Groups

The UTR evaluation was carried out on 13 July, 23 August, and 25 October 2024 and 16 January 2025.

Pregnancy and PPG diagnoses were made using B-mode transabdominal ultrasonography (UTR) between 40 and 60 days after the bucks’ removal (BR) from pens. The UTR uterus assessment was performed by the same operator in all the 605 goats using a portable scanner (Iberscan A9^®^; CCPA, Janzé, France) with a convex multifrequency probe (2.5, 3.5, and 5.0 MHz). Goats were kept in a standing position, and the transducer was positioned on the inguinal area across the cranial abdomen to the pelvic brim [11].

A PPG diagnosis is made based on the presence of an enlarged intrauterine anechoic area, meaning free fluid and hyperechoic lines representing the juxtaposition of uterine wall folds, without the presence of an embryo or fetus. These traits correspond to those categorized as grade 3 and 4 (scale: 0 to 4) reported by [6] for transrectal UTR using a 5.0 MHz transducer. Positive and negative pregnancy diagnoses were made based on the presence and absence of fetal heartbeats or fetus, respectively [11]. The kidding was confirmed in all PG does. PPG does were treated and moved with NPG does to the next group for re-breeding.

A total of 47 does were classified as the PPG group. For group formation and blood sampling purposes, a total of 10 pregnant (PG; positive control group) and 11 nonpregnant (NPG; negative control group) does were randomly selected. When one or more PPG does were found, at least one pregnant doe and one nonpregnant doe (about 4% of non-PPG does) were sampled at each farm visit for the UTR session. This was a randomized controlled trial after the stratification of NPG and PG does by parity to avoid potential bias regarding this factor [24].

The parity of the does was recorded based on evidence [24] suggesting that it can affect plasma PAG levels. Moreover, the kidding–UTR interval, BI-UTR interval, and BR-UTR interval of each doe were systematically registered to assess the timeline patterns of PAG production by does.

### 2.3. Sampling and Laboratory Evaluation of Hormones

Blood samples were collected through jugular venopuncture to a dry tube at the time of diagnosis and sent to a commercial accredited laboratory (Vetdiagnos, Diagnóstico Veterinário^®^, Cantanhede, Portugal) in the following four hours under refrigeration (at approximately 4 °C). Plasma was separated with centrifugation (2000 rpm; 15 min) and samples were stored at −20 °C until the end of the study period.

P4 plasma levels were obtained through electrochemiluminescence immunoassay using Elecsys Progesterone III^®^ (Roche Diagnostics, Mannheim, Germany) [25,26]. A cut-off of 0.1 ng/mL of P4 was used to determine the presence or absence (<0.1 ng/mL) of active CL [27].

For PAG determination, an enzymatic immunoassay with a sensitivity of 100% and specificity of 91.4% (Alertys Ruminant Pregnancy Test^®^, Idexx, Hoofddorp, The Netherlands) was used according to the manufacturer’s instructions [28,29]. In this assay type, the correction (S-N) of the optical density (OD) is performed by using the OD subtraction of the sample (S) from the OD of the negative control (N), measured with a 450 nm wavelength. The S-N cut-off value of ≥0.3 optical density (OD) is used to detect pregnancy [28,30]. The results of the plasma PAG evaluation are presented as S-N OD units (subsequently referred to as OD) and the PAG/P4 ratio as OD (PAGs)/ng/mL (P4).

Plasma or serum PAG evaluation can be used as early as 28 days after breeding in goats, without interference from a previous pregnancy [2,25].

### 2.4. Statistical Analysis

All continuous variables were evaluated for normality distribution of residuals using the Shapiro–Wilk test. PAGs and PAG/P4 ratio were Box–Cox-transformed for comparison between the groups. One-way ANOVA and Tukey HSD were applied to test differences in parity, kidding–UTR interval, BI-UTR interval, and BR-UTR interval between the groups. Pearson correlations were used between continuous variables.

Logistic regression models followed by receiver operating characteristic (ROC) analysis were used to determine the sensitivity and specificity of biomarkers for PPG, NPG, and PG diagnostics. The thresholds of the PAG/P4 ratio, PAGs, and P4 were assessed using Youden’s index [sensitivity − (1 − specificity)], provided by ROC tables. The positive (PPV) and negative (NPV) predictive values of the PAG/P4 ratio were determined by the following equations: PPV = [TP/(TP + FP)] × 100 and NPV = [TN/(TN + FN)] × 100.

JMP^®^ version Pro 16 statistical software was used for all analysis, including the retrospective evaluation of statistical power, i.e., post hoc power analysis, for PAGs, P4, and PAG/P4 inferential analysis. For this purpose, σ (residual error) and δ (effect size) values were evaluated according to [31] and for α = 0.05. Adjusted statistical power values were also determined to remove positive bias and for a 95% confidence interval.

The results are presented as least-squares mean ± SEM for a 0.05 level of significance.

## 3. Results

The incidence of PPG observed in this study was 7.8% (47/605; 95% confidence interval: 5.9 to 10.2%). No significant differences (*p* = 0.50) in mean parity were observed between the PPG (2.3 ± 0.2; n = 47), NPG (2.6 ± 0.4; n = 11), and PG (1.9 ± 0.5; n = 10) groups.

We observed a tendency (*p* = 0.08) for a higher kidding–UTR interval in PPG (599.6 ± 56.1 days) than in NPG (346.4 ± 114.8 days) or PG (399.6 ± 56.1 days) (n = 10) does. The BI-UTR interval was similar (*p* = 0.92) between the PPG (76.8 ± 1.5 days), NPG (75.8 ± 3.0 days), and PG (77.5 ± 3.1 days) groups. Furthermore, no significant differences (*p* = 0.52) in the BR-UTR interval were observed between PPG (34.5 ± 0.5 days), NPG (34.1 ± 1.0 days), and PG (33.2 ± 1.0 days) does.

### 3.1. Hormone Profile

A significant effect (*p* < 0.001) of reproductive status on plasma PAG OD values was observed between groups, with PG does presenting the highest levels (Table 1). The range of PAGs was 0.05 to 0.28 OD in PPG does. Lower P4 levels were observed in NPG does than in PG or PPG does (*p* < 0.001), but they were similar between the PG and PPG groups. The range of P4 was 1.3 to 18.1 ng/mL in PPG does. Plasmatic P4 levels ≥ 1.0 ng/mL were observed in 27.3% (3/11) NPG does.

The correlation between PAGs and P4 for pregnant goats was r = 0.78 (r^2^ = 0.61; RMSE = 0.56; n = 10; *p* < 0.01). No significant correlations between these two variables were observed for the NPG (*p* = 0.94) and PPG (*p* = 0.20) groups.

A negative correlation r = −0.69 (r^2^ = 0.47, RMSE = 0.06; n = 11; *p* < 0.05) between the BI-UTR interval and serum PAG levels was observed in the NP animals. No other correlation (*p* > 0.05) was observed for PAGs, P4, or PAG/P4 ratio, and this interval was observed.

### 3.2. PAG/P4 Ratio Biomarker

The logistic regression model regarding the UTR diagnosis of the goats’ reproductive status was significant (*p* < 0.001).

**Figure 1:**
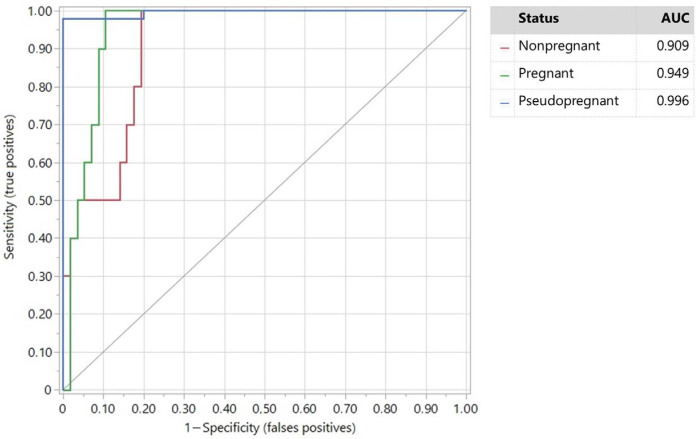
ROC plot of the PAG/P4 ratio based on pseudopregnant, nonpregnant, and pregnant does.

The ROC curve used to estimate the sensitivity and specificity of the PAG/P4 ratio according to the UTR examination is presented in Figure 1. For PPG diagnosis, the sensitivity was 97.9% for a specificity of 100%; if the sensitivity of the PAG/P4 ratio increased to 100%, the specificity decreased to 80%. The sensitivity was 100% in PG and NPG dairy does for a specificity of 89.3% and 80.8%, respectively. To reach a specificity of 100%, the sensitivity was 89.0% and 79.6% in PG and NPG dairy does, respectively.

According to Youden’s index, the best cut-off of the PAG/P4 ratio value to consider a positive diagnosis of PPG was 0.044.

The PPV and NPV values of the PAG/P4 ratio were 100% and 95.2%, respectively.

**Figure 2:**
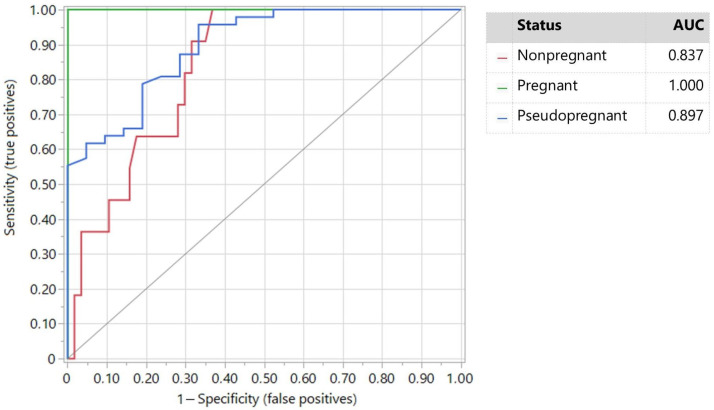
ROC plot of PAGs based on pseudopregnant, nonpregnant, and pregnant does.

**Figure 3:**
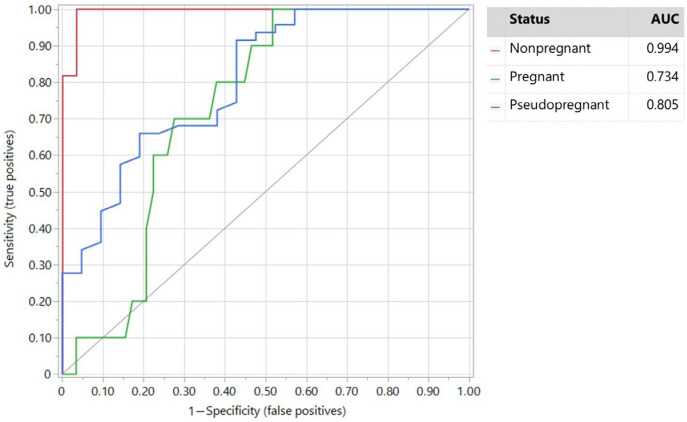
ROC plot of P4 based on pseudopregnant, nonpregnant, and pregnant does.

The accuracy of PAGs and P4 in diagnosing PPG was lower than the PAG/P4 ratio biomarker. The AUCs were 0.897 and 0.805 for PAGs and P4, respectively. PAGs remained the best predictor for diagnosing PG does (Figure 2) and P4 (Figure 3) for diagnosing NPG does when all three levels were analyzed together.

According to Youden’s index, the best cut-off value of PAGs to diagnose pseudopregnancy was 0.175 OD for a sensitivity of 95.7% and specificity of 66.6% (AUC = 0.897).

For P4, the best threshold to diagnose PPG was 2.17 ng/mL for a sensitivity of 95.7% and specificity of 53.4% (AUC = 0.676).

## 4. Discussion

Pseudopregnancy is a reproductive disorder affecting fertility and productivity in dairy goats, with reported mean incidences of less than 9–10% [7,8,14,32], which is in agreement with the observed value in our study (7.8%). However, some studies recorded variations between 3.0% [7,14,32] and 54% [3]. Lopes Júnior et al. [33] found a PPG prevalence of 30.4% in Saanen goats, much higher than the value reported in our study for the same breed. The different rates of pseudopregnancy documented in these different studies may be understood by considering factors such as a higher dairy-breed-specific susceptibility [1,34,35]. Environmental and geographical influences, or even differences in reproductive management practices, can also contribute to high PPG prevalences. As an example, in some breeds, such as Saanen, a higher milk production and persistence of lactational curve under certain management strategies can be an advantage [36]. However, the use of extended lactation as a management practice seems to favor the incidence of PPG [3]. This is also suggested in our study by the large kidding–UTR interval, mainly in the PPG group.

Although not statistically significant, a tendency for a longer kidding–UTR interval, 200 or more days, was observed in animals with PPG compared to NP or PG does, in accordance with other authors [3]. This period coincides with days in lactation and therefore an extended generational interval, confirming the role of PPG as a cause of infertility and productivity in animals (when reared for meat and milk, i.e., dual-purpose breeds).

In our study, the mean plasma PAG level was 1.45 ± 0.04 OD in the PG group diagnosed between about >1 and 2.5 months after mating. This concentration was at least 10 times higher than those of the other two groups. Plasma PAG levels are a pregnancy and trophoblastic well-being indicator [37]. In goats, a threshold of 1.5 ng/mL, measured using radioimmunoassay, was considered to determine the occurrence of pregnancy [17]. Nonetheless, for the ELISA test used in our study, a cut-off of 0.3 OD is recommended by the manufacturer [28] and confirmed by [29] in beef cattle (0.26 ± 0.036 OD). Using the (IDEXX) ELISA test, the authors of [30] observed that the plasma PAG levels in PG Jakhrana goats were 2.14 ± 0.40 OD at day 51 of pregnancy. In NPG goats, this level was 0.06 ± 0.03 OD. Moreover, plasma PAG levels of 0.42 ± 0.12 OD and 0.09 ± 0.03 OD at day 35 after artificial insemination were observed in PG and NPG East Friesian ewes [38]. At day 56 after mating, the serum PAG concentration was 0.80 ± 0.12 OD in Awassi ewes, whose levels increased from 0.24 ± 0.03 OD at day 28 [39]. More recently, this research group observed that the mean serum PAG level, in the first 11 weeks post mating, was 0.51 ± 0.04 OD for Karya and Konya Merino ewes, with a significant (*p* < 0.001) time effect [40]. The PAG profiles of these local breeds were similar to those observed in the PG and NPG groups in our study.

No data on plasma PAG levels regarding PPG in goats or other species were found in the scientific literature. According to our results, PAG values below the 0.3 OD threshold cannot differentiate, per se, PPG from NPG does consistently, with even the PAG levels being different between them. The mean difference in PAG levels between the PPG and NPG groups was low (0.5 OD), and none of the field conditions of breeding, including its seasonality, were fully addressed in this study. The low specificity (66.6%) observed for the PAG biomarker for diagnosing PPG considering both NPG and PG does (see Figure 2) also supports this interpretation.

Since the BI-UTR interval was 76.8 ± 1.5 days, and since after embryonic and/or fetal viability, or kidding, the blood levels of PAGs decrease more quickly in goats than in cows [17,41], it was also not possible to obtain information about potential pregnancy loss in our study. A mean OD of 0.11 ± 0.06 was observed in ewes presenting late embryonic mortality at day 35 after artificial insemination, evidencing the quick PAG decrease after pregnancy loss [38]. However, no similar information was retrieved in the main scientific databases for goat species, and further research to cover this gap is required.

In our study, a negative correlation (r = −0.69) between the BI-UTR interval (average of 75.8 ± 3.0 days) and plasma PAG levels were observed in the NPG group. The kidding–UTR interval was 346.4 ± 114.8 days, and the BR-UTR interval was 34.1 ± 1.0 days in this group, and it was reported that the serum PAGs’ half-life in goats is approximately 7.5 days, with complete elimination between 14 and 28 days post kidding [2]. Nonetheless, there is a possibility that higher PAG values, but always <0.3 OD, can be due to potential embryonic death in some NPG goats, for no apparent or known reasons. This is an open question that can be answered with early and successive UTR examinations in further research.

The plasma P4 levels were higher in the PPG group than in the NPG group, confirming the presence of a functional CL as a typical condition for this disease [42]. A P4 blood measurement of ≤0.1 ng/mL accurately detects nonpregnancy in early gestation [43,44,45]. However, above this threshold, the P4 values were not enough to differentiate PPG from normal pregnancy (see Table 1). In accordance with our findings, Llewelyn et al., 1992 [42], observed similar P4 profiles for pregnancy and PPG during the first 80 days of pregnancy. During the breeding season (September to January), Saanen goats and their crossbreeding with similar strong seasonal genotypes (e.g., Alpine bucks) are cycling, but their anestrus persists during the non-breeding season, even in the latitudes that we studied (<45°). This reproductive behavior can justify the high variance of P4 levels observed in NPG does, as well as the 27.3% of does with an active CL, i.e., in the diestrus phase of the estrous cycle.

The high and significant correlation between the plasma levels of P4 and PAG hormones in the PG group does indicate a close relationship during pregnancy, likely due to their production by the placenta and CL, respectively. PAG levels may positively impact serum P4 levels, and in vitro research has revealed that the administration of PAGs to luteal cell cultures may increase the progesterone output by these cells [46]. Although a strong positive association between P4 and PAGs was observed in Barbari goats by [47], these hormones have a distinct temporal pattern, with PAG concentrations reaching a peak earlier in gestation (8 weeks) than P4 (10–14 weeks). In goats with failed pregnancy, the PAG concentration starts to decline 12 days before the P4 concentration does [30].

The high sensitivity and specificity of the PAG/P4 ratio observed in our study, using UTR as a gold-standard method, for pseudopregnancy diagnosis associated with an AUC = 1 confirms that this ratio can be used as a biomarker. This biomarker may be useful for dubious situations associated with early pregnancies or depending on the veterinarian’s experience. The lower PAG/P4 ratio in the PPG group observed in our study was mainly due to different combinations of each hormone according to the reproductive status of the does. PPG does exhibited low and high levels of PAGs and P4, respectively; these levels were both high in PG does. This allows us to establish the PAG/P4 ratio as an accurate biomarker for diagnosing PPG with a high sensitivity and specificity regarding the positive control group, as reported in Figure 1. In NPG does, both plasma PAGs and P4 remained lower on average but overtook the low cut-off value (0.044) for the PAG/P4 ratio. Since (1) the serum or plasmatic P4 levels in PPG, pregnancy, or diestrus, by definition, present an active CL ≥ 1 ng/mL, and (2) the cut-off for PAGs only is low (>0.3 DO) in PPG and NPG does, a low number of false negative or false positive PPG does can surge, if the UTR diagnosis is made more than about 30 days after kidding or mating. These false negatives are probably mainly due to cycling goats in diestrus or does eventually presenting with any other disease with persistence of CL or luteal tissue (e.g., luteinized ovarian cysts) [48]. PPG false positives can occur if the goat is diagnosed shortly after giving birth, when PAG levels are still high and the PAG/P4 ratio may fall below the cut-off value, or if embryonic mortality occurs less than 25 days before blood is sampled [45,49].

Finally, the findings of this study fully support our initially proposed hypothesis. The high PPV and NPV values of the PAG/P4 ratio indicate the feasibility of this biomarker for diagnosing PPG. Nonetheless, some limitations of this study have been identified, which were mainly due to the intrinsic breeding management of the four farms and to the suitability of the study design for this management. (a) The UTR evaluation day was different between goats; (b) no early diagnosis, e.g., within 21 days after mating, was performed (e.g., using transrectal UTR); (c) there was a lack of successive sampling; and (d) a fully non-breeding season was not considered. Moreover, indirect determination of embryonic death and timeline determination of PAGs are two outcomes regarding their relevance for etiology, diagnosis, and prevention of PPG. Further research in this direction is needed to support clinical decisions relating to this disease. Although a strong statistical power to evaluate the biomarkers was observed in this study, a large sample is required to confirm the influence of some factors, such as parity and kidding–UTR intervals, with regard to PPG prevalence. Furthermore, the PAG/P4 ratio biomarker should be clinically tested in a large population comprising different breeding management approaches.

## 5. Conclusions

In this study, we detected significant differences in hormone levels across reproductive statuses, with notable increases in PAGs during pregnancy, elevated P4 during pregnancy and pseudopregnancy, and significant differences in the PAG/P4 ratio. These differences are highly statistically significant, supporting their potential use as biomarkers for reproductive status.

The PAG/P4 ratio seems to be an accurate biomarker to serve as an alternative to UTR or even to complement the latter methodology in veterinary assistance to farms. More clinical research is required to evaluate this ratio under several management conditions and even for other fluids such as milk.

## Figures and Tables

**Table 1:** LS mean ± SEM of pregnancy-associated glycoproteins (PAGs), progesterone (P4), and PAG/P4 ratio according to the reproductive status of dairy goats.

Hormone	Reproductive Status			*p*-Value	Statistical Power (α, σ, δ)
	PPG (n = 47)	NPG (n = 11)	PG (n = 10)		
PAGs (OD)	0.08 ± 0.02 ^a^*	0.13 ± 0.04 ^b^*	1.45 ± 0.04 ^c^	<0.001	1 ^╪^ (0.05, 0.13, 0.32)
P4 (ng/mL)	6.76 ± 0.49 ^a^	0.69 ± 1.00 ^b^	8.15 ± 1.05 ^a^	<0.001	0.9997 ^╪^ (0.05, 3.32, 2.38)
PAG/P4 ratio	0.01 ± 0.11 ^a^	0.24 ± 0.23 ^b^	0.18 ± 0.23 ^b^	<0.001	0.9931 ^╪^ (0.05, 0.72, 1.25)

PPG: pseudopregnancy; NPG: nonpregnancy; PG: pregnancy. OD: optical density (ELISA). ^a–c^ different superscript letters in the same line: *p* < 0.001, except for *: *p* =0.002. ^╪^ Adjusted statistical power and respective 95% confidence interval for PAGs (1; 95% CI: 1–1), P4 (0.9993; 95% CI: 0.8510–1), and PAG/P4 ratio (0.9856; 95% CI: 0.5349–1).

## Data Availability

The data that support the findings of this study are available on request from the corresponding author.
